# Object recognition along the ventral stream: effects of visual noise

**DOI:** 10.64898/2026.02.10.705069

**Published:** 2026-02-11

**Authors:** Ajay Subramanian, Ekin Tünçok, Jan W. Kurzawski, Najib J. Majaj, Denis G. Pelli, Jonathan Winawer

**Affiliations:** 1 Department of Psychology, New York University, New York, NY, USA; 2 Department of Biomedical Engineering, Columbia University, New York, NY, USA; 3 Faculty of Psychology and Neuroscience, Maastricht University, Maastricht, Netherlands; 4 Center for Neural Science, New York University, New York, NY, USA; 5 Lead contact

**Keywords:** object recognition, spatial frequency, visual cortex, ventral stream, fMRI

## Abstract

Psychophysical studies using critical-band masking have established that human object recognition is most impaired by stimulus noise in a narrow, 1.5 octave band of spatial frequency. This shows that visual recognition relies on this narrow band. Here, we investigate the biological basis of this recognition band. We used fMRI to measure BOLD responses along the ventral stream (V1 → V2 → V3 → V4 → ventral temporal cortex, VTC) to band-pass noise and to natural images perturbed by that noise. The response to noise alone widens from 2 octaves in V1 to 5 octaves in VTC, diverging more and more from the behavioral 1.5 octave recognition bandwidth. In contrast, a correlation classifier applied to the BOLD response to noisy images shows that the recognition bandwidth is conserved at 2 octaves. This contrast shows that the recognition band is more like a readout constraint than an early sensory filter. Furthermore, this 2-octave band becomes increasingly tolerant to noise, approaching behavioral tolerance in VTC. Thus, it seems that recognition improves along the ventral stream by maintaining a narrow object channel while increasing noise tolerance.

## INTRODUCTION

We recognize objects despite the variability of our natural environment and the inherent noise in our visual system. What makes human object recognition robust to noise? Psychophysical studies suggest that one way that vision accomplishes this is by using a narrow-band *channel* for recognition. Psychophysically, critical-band masking (Fletcher, 1940) experiments indicate that people recognize gratings (Campbell and Robson, 1968), letters (Majaj et al., 2002, Solomon and Pelli, 1994), and natural objects (Subramanian et al., 2023) using a 1.5 octave-wide spatial-frequency channel. Importantly, the narrowness of this channel is a property of the visual system, not of the stimulus (Figure S1). Noise confined to this narrow frequency band impairs recognition, whereas noise outside it has little effect. By comparison, deep-learning-based vision systems tend to have much wider recognition channels, around 4 octaves wide (Subramanian et al., 2023). The width of their channel is negatively correlated with measures of recognition robustness (Subramanian et al., 2023): shape bias (Geirhos et al., 2018a), and resistance to adversarial attacks (Szegedy et al., 2013). Here, we investigate the biological basis, in humans, of the narrow channel for object recognition.

**Figure 1:**
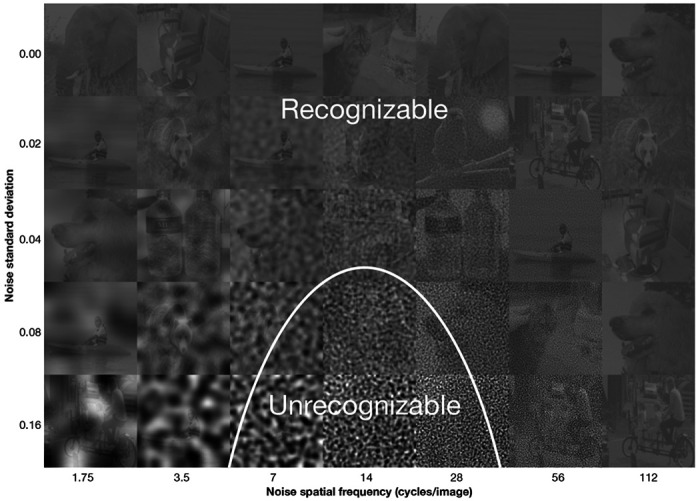
Critical-band masking and the 1.5 octave psychophysical object recognition channel. A demo of how critical-band masking reveals a spatial-frequency channel for object recognition. Each cell in the grid contains a sample grayscale, contrast-reduced (20%) image from ImageNet, perturbed with filtered Gaussian noise. Rows correspond to increasing noise standard deviation (σ) from top to bottom. Columns correspond to noise filtered within one-octave spatial-frequency bands centered at increasing frequencies from left to right. To visualize the channel, determine how far down each column you can go before being unable to recognize the object. These thresholds trace out an inverted-U-shape like curve shown in white, typically centered at 14 cycles/image, identifying the spatial frequencies most critical for recognition. The plot displays the noise threshold SD predicted by our spatial-frequency channel model as a function of spatial frequency, mirroring the perceptual sensitivity. Curve has been shifted to the left by half an octave for the demo, to account for the small size of the images in the printed demo compared to the experiment, based on previous studies of scale dependency (Majaj et al., 2002).

We use critical-band masking to study the effect of stimulus noise on neural representations along the human recognition pathway: V1 → V2 → V3 → V4 → ventral temporal cortex (VTC) (Mishkin et al., 1983). The main idea is that the spatial frequencies critical for recognition are the ones where added noise most disrupts recognition; masking those frequencies causes recognition to fail (Figure 1). Previous studies (Campbell and Robson, 1968, Majaj et al., 2002, Solomon and Pelli, 1994, Subramanian et al., 2023) have applied this method to study behavioral responses. In this paper, we apply it to the study of neural representations.

We measure BOLD responses to band-pass noise images and to natural images perturbed by that noise (Figure S2). We define two distinct neural metrics: The amplitude of the BOLD response to noise alone defines a *noise response band* and the decoding accuracy of the BOLD response to noisy images defines a *recognition band*. Our goal is to determine how the width of each band and its tolerance to noise evolve along the ventral pathway and how they relate to behavioral object recognition.

## RESULTS

### 1. The response to noise broadens and diminishes along the ventral stream.

To characterize the tuning of visual cortex to noise, we measured BOLD responses to band-pass noise stimuli in the absence of scenes. We fit a spatial-frequency channel model to these responses separately for each visual area.

**Figure 2:**
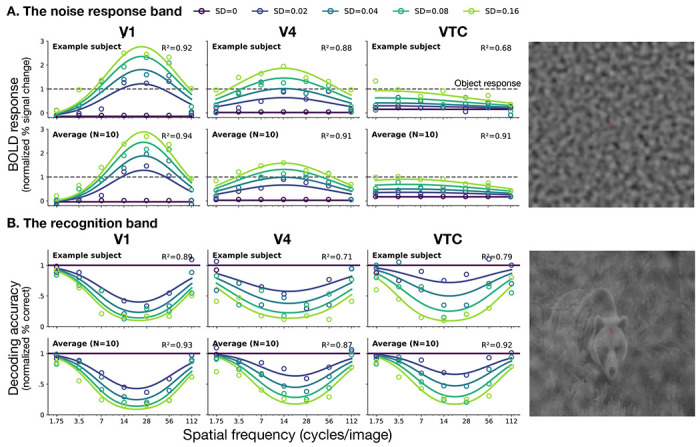
Divergent tuning of noise response and object recognition bands along the ventral stream. **A. The noise response band.** BOLD response amplitude (% BOLD signal change) to band-pass noise alone, normalized by scene-alone response, as a function of spatial frequency. Rows show data and fits for an example observer (top) and the average across observers (bottom) in areas V1, V4, and VTC. Color indicates noise standard deviation (σ). Dashed horizontal lines represent the response amplitude to the scene alone in each area (=1, normalized). **B. The recognition band.** Accuracy of object decoding from noisy images, normalized by decoding accuracy for zero-noise images, as a function of noise spatial frequency. Rows correspond to the individual (top) and average (bottom) observer for regions V1, V4, and VTC. Solid horizontal lines indicate baseline decoding accuracy for noiseless images (σ = 0). Circles represent measured data points; solid curves represent predictions from the fitted spatial-frequency channel models, with *R*^2^ values indicating goodness-of-fit for each ROI. The images on the right shows a sample stimulus from the experiment used to measure each band.

The noise response diminishes from V1 to VTC, its bandwidth broadens, and its peak shifts to lower frequencies (Figure 2A). Figure S4 shows noise response bands for all observers in all visual areas. V1 responds most strongly to noise at 19 cycles/image, declining sharply for lower and higher frequencies, with a 2-octave bandwidth. V4 responds moderately to noise, peaking at a lower frequency (14 cycles/image) with a wider bandwidth (4 octaves). VTC responds only weakly to noise and its tuning is low pass. Comparing these responses to the response amplitude to natural images (dashed lines), we see a shift from preferring noise in V1 to preferring images of natural scenes in VTC.

### 2. Unlike the response band, recognition bandwidth is conserved along the ventral stream.

To determine whether the broadening of the response to noise implies a broadening of the effect of the noise on recognition, we applied an image classifier to the BOLD response to noisy images. This yielded a recognition band for each visual area (Figure 2B). Figure S5 shows recognition bands for all observers in all visual areas. Also, the patterns in noise response and recognition bands are both conserved across eccentricity (Figure S7, S8).

In contrast to the noise response band, the recognition band is conserved along the ventral stream: The decoding accuracy curves for V1, V4, and VTC all exhibit a similar narrow, inverted-U shape, about 2 octaves wide. Because the recognition band is conserved and the noise response band is not, the two bands diverge more and more. For example in V1, the effect of noise on decoding is approximately mirror-symmetric to the response to noise alone, whereas in VTC the two patterns are strikingly different.

**Figure 3:**
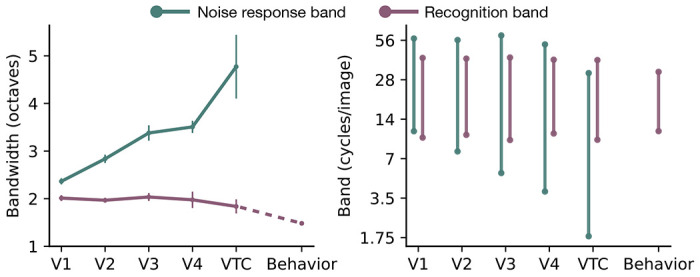
Divergence between noise response and recognition bands. **Left:** Channel bandwidths are plotted for visual areas V1 through VTC, and behavior. **Green line** shows the bandwidth of the noise response band (defined as the FWHM of the fitted Gaussian tuning), which widens progressively from 2 octaves in V1 to 5 octaves in VTC. **Purple line** shows the bandwidth of the recognition band (defined as the width at twice the minimum power threshold), which remains conserved at 2 octaves in all areas, close to the behavioral 1.5 octave recognition bandwidth. **Right:** Range of frequencies within each band for each visual area. Noise response band grows wider and shifts leftward from V1 to VTC. Recognition bandwidth and center frequency stay conserved from V1 to VTC at values similar to the behavioral recognition band. For behavior, the error bar is smaller than the dot size. Data points represent the mean across subjects (*N* = 10 for fMRI, *N* = 14 for psychophysics), and error bars indicate ±1 SEM. bring dot size down, add a dashed line.

We summarize the divergence by calculating the bandwidth of the noise response band and the recognition band for each area (Figure 3, left). The noise response bandwidth increases monotonically from 2 octaves in V1 to 5 octaves in VTC. In contrast, the recognition bandwidth is conserved at 2 octaves, close to the 1.5 octave band measured psychophysically. The noise response band grows wider and shifts leftward from V1 to VTC whereas the recognition band maintains a 2-octave bandwidth and a 19 cycles/image preferred frequency across all areas, mirroring the behavioral recognition band (Figure 3, right).

### 3. Along the ventral stream, noise response becomes weaker and recognition becomes more noise-tolerant.

We quantify *noise threshold* as the noise power that elicits a BOLD response equal to that of the scene alone. We quantify *noise tolerance* as the noise power required to reduce the decoding accuracy by half.

**Figure 4:**
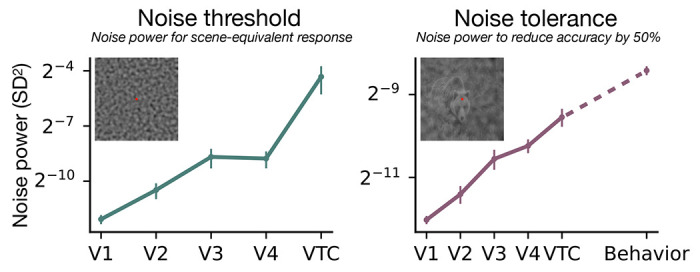
Both the noise threshold and tolerance increase along the ventral stream. **Left: Green line** shows the noise power *σ*^2^ required to produce peak response equal to the response to scene alone, in each visual area. Later areas require more noise to elicit scene-equivalent response, indicating higher signal-to-noise ratio (SNR). **Right: Purple line** shows the noise power required to reduce decoding accuracy to 50% of the noise-free baseline for each visual area. Noise tolerance of object decoding increases from V1 to VTC, approaching the tolerance of behavioral object recognition. This indicates that high-level object representations are robust to noise levels that disrupt early visual areas. For behavior, the error bar is smaller than the dot size. Data points represent the mean across subjects (*N* = 10 for fMRI, *N* = 14 for psychophysics), and error bars indicate ±1 SEM. same changes as fig 3.

Both the noise threshold and tolerance increase along the ventral stream (Figure 4). The noise threshold (green line) rises steeply from V1 to VTC, a 64:1 increase, indicating that downstream areas require more noise power to reach a comparable activation level as early areas. Because we define the noise threshold relative to the response to scene alone, an increasing noise threshold could be due to a reduced noise response or an increased scene response. Looking at the raw responses along the ventral stream, the response to scene alone is conserved and to noise alone drops (Figure S6). This suggests that successive areas denoise the input.

The recognition band follows the same trend. The noise tolerance increases 8-fold from V1 to VTC (purple line), indicating that high-level representations are more robust to noise. In VTC, the noise tolerance of image decoding approaches that of behavioral recognition (dashed black line).

## DISCUSSION

Our results reveal how the visual system manages noise along the ventral stream. First, as we move along the ventral stream, higher areas respond less to noise, enabling more noise-tolerant image decoding. Second, the spatial-frequency bandwidth of the noise that drives the BOLD response broadens dramatically from V1 to VTC, while the bandwidth of the noise that impairs image decoding remains narrow.

Consistent with Ha et al. (2025), we find that neural tuning to noise broadens downstream; though see Aghajari et al. (2020). However, these studies did not evaluate recognition. Here, using critical-band masking, we reveal that the recognition bandwidth is conserved unlike the noise response band.

### The narrow tuning of the recognition band does not result from linear filtering

The similarity of recognition and noise response bands in V1 might suggest simple linear filtering, but the responses in the rest of the ventral stream, especially VTC, contradict this: In VTC, the noise response band is low-pass while the recognition band remains band-pass. We confirmed the rejection of linearity by a superposition test (Figure S3). Unlike V1, where noise and scene responses are additive, in VTC, the noise response is strongly suppressed by the scene. This suppression – which emerges gradually along the ventral stream – is strongest at mid-frequencies (Figure S3), i.e., the critical recognition band.

Since VTC encodes noise across 5 octaves while only 2 are diagnostic, the recognition band likely functions as a readout constraint that filters available neural information. This is consistent with macaque IT studies showing that linear readouts can be trained to select specific features for specific tasks (Hung et al., 2005, Rust and DiCarlo, 2010) and fMRI evidence that behavioral goals modulate representations (Harel et al., 2014).

This strategy is notably sub-optimal in an information-theoretic sense. An “ideal observer” would utilize all available spatial frequencies to maximize the signal-to-noise ratio (Geisler, 2011, Neri, 2015). For many tasks, deep neural networks, like ideal observers, use a broad range of frequencies; However, this broad tuning makes them brittle (Subramanian et al., 2023). By strictly limiting the readout to a narrow band, the human visual system likely sacrifices information to achieve greater robustness across varying environments.

### Implications for artificial vision: invariance versus untangling

These findings highlight a divergence between human vision and deep neural networks. Our data support the idea that human vision achieves robustness through *untangling* (DiCarlo and Cox, 2007). VTC retains a strong representation of the noise — evidenced by the broad, 5-octave noise response band — but minimizes the noise’s effect on recognition. The brain does not suppress the “fog” to see the “car”; it represents both. While most CNNs lack the narrow recognition channel of human vision (Subramanian et al., 2023), recent foundation models aligned with language like CLIP (Radford et al., 2021), and adversarially trained networks (Madry et al., 2017) show improved robustness to noise. The improved robustness might make them more similar to human behavior, but they likely achieve this through a mechanism distinct from the human brain. We hypothesize that current robust models rely on *invariance* rather than untangling. Their training objectives — whether through contrastive learning or adversarial defense — explicitly force the model to map a noisy image to the same point in feature space as its noise-free counterpart. This pressure drives the network to suppress noise entirely, effectively making the system blind to the perturbation (Chen et al., 2020, Madry et al., 2017). To build truly brain-like models, we should not aim for systems that are blind to noise, but rather that can separate object from noise.

### Why does noise tolerance increase along the ventral stream?

Object decoders in VTC tolerate 8 times more noise power than those in V1. Contrary to our initial expectation, the recognition band is no narrower in VTC than in V1, so the increase in tolerance cannot be due to narrowing bandwidth. Perhaps higher noise tolerance arises from the sensitivity of higher areas to higher-order image statistics. V1 is responsive to localized contrast energy whereas in V2 and beyond, the responses are dependent on higher-order statistics, such as correlations between outputs of different localized, linear filters (e.g., ziemba, but need other papers) (Ziemba et al., 2016). Presumably these higher-order statistics are better and better at separating object from noise (Lieber et al., 2024).

## CONCLUSION

Perhaps the most surprising result is the divergence between how the brain responds to noise alone and how noise affects the response to scenes. The noise response bandwidth grows (from 2 to 5 octaves) along the ventral stream becoming less and less like the behavioral bandwidth (1.5 octaves). On the other hand, the recognition bandwidth is similar to the behavioral bandwidth and conserved along the ventral stream. We also discovered that the tolerance of image decoding to noise improves 8-fold along the ventral stream. Thus, it seems that recognition improves along the ventral stream by maintaining a narrow object channel while increasing noise tolerance.

## RESOURCE AVAILABILITY

### Lead contact

Requests for further information and resources should be directed to Ajay Subramanian (ajay.subramanian@nyu.edu).

### Materials availability

This study did not generate new materials.

## METHODS

### 1 EXPERIMENTAL MODEL AND STUDY PARTICIPANT DETAILS

#### 1.1 Participants

Data were collected from 10 healthy human volunteers (5 female, 5 male). All participants had normal or corrected-to-normal visual acuity. Participants provided written informed consent in accordance with the experimental protocol approved by the New York University Institutional Review Board. Age, sex, and gender were not considered as variables in the study design.

### 2 METHOD DETAILS

#### 2.1 Apparatus

Experimental stimuli were generated on an Apple iMac MATLAB (R2023) and Psychtoolbox-3 (Kleiner et al., 2007) and displayed on a ProPixx DLP LED projecter (VPixx Technologies Inc., Saint-Bruno-de-Montarville, QC, Canada). Participants viewed the projected screen (60 x 36.2 cm; 1920 x 1080 resolution; refresh rate of 60 Hz) through an angled mirror mounted on the scanner’s head coil. The viewing distance from the eye to the mirror to the screen was 86.5 cm. Participant responses were collected using a 4-button fiber optic response box (Current Designs). Participants could use any of the four buttons to respond. Head pads were used inside the coil to stabilize participants head and reduce head motion and discomfort.

#### 2.2 Stimuli

Stimuli for the main experiment were generated using the same image set and preprocessing pipeline as Subramanian et al. (2023). Briefly, we started from grayscale versions of natural images drawn from the 16-class ImageNet subset (Deng et al., 2009, Geirhos et al., 2018b) and selected 10 exemplar images from 10 distinct superordinate categories (bird, dog, cat, bear, elephant, truck, boat, chair, bottle, bicycle). Images were first resized to 256 × 256 pixels, center-cropped to 224 × 224 pixels (the standard ImageNet preprocessing protocol), and then upsampled to 512 × 512 pixels for presentation. All images were converted to grayscale and normalized to have low contrast by scaling their pixel intensities to 20% of their original root-mean-square (RMS) contrast about a midgray level (intensity 0.449 on a [0,1] scale). This ensured that adding high-strength noise would not produce substantial pixel clipping at floor or ceiling.

Band-pass noise was added to these low-contrast images following the critical-band masking procedure described in Subramanian et al. (2023). On each trial, we sampled a Gaussian white-noise field and, for non-zero noise conditions, filtered it into one of seven octave-wide spatial-frequency bands using a Laplacian pyramid decomposition (Burt and Adelson, 1987). The seven bands corresponded to successive levels of the pyramid and were approximately centered at 0.875, 1.75, 3.5, 7, 14, 28 and 56 cycles per image, each spanning one octave (a factor of two in spatial frequency). For each band, the filtered noise was rescaled so that its RMS contrast matched a desired noise strength and then added to the low-contrast scene. We used five noise strengths (noise standard deviations of 0, 0.02, 0.04, 0.08, and 0.16, in units of normalized image intensity). The zero-noise condition (SD = 0) yielded low-contrast images without added noise. After addition of noise, pixel values were clipped to the [0,1] range if necessary.

In addition to noise-masked scenes, we also generated noise-only stimuli by adding band-pass noise (constructed as above) to a uniform midgray image (intensity 0.449). This produced a matched set of band-pass noise patterns at each spatial frequency and noise strength, without any overlaid scenes. All stimuli were presented centrally on a uniform gray background, and the same set of object and noise conditions was used across all fMRI runs.

#### 2.3 Experimental Protocols

##### Main experiment

Participants completed 11, 13 or 15 scans of the main event-related fMRI experiment (Table 1). Each image, created as described above, was presented for 3 sec with 1 sec off time between two consecutive image presentations. We adapted this protocol from previous work that used event-related presentation of natural images and elicited reliable responses from visual cortex (Allen et al., 2022). Participants viewed 87 images on each scan session, totaling up to the following numbers of total image presentation across the experiment:

Throughout the experiment, participants were asked to fixate at the center of the display. They performed a color change detection task at the fixation dot by making a button press. Each scan lasted 6 min 36 seconds (396 TRs).

##### Retinotopic mapping

Participants completed six scans of a retinotopic mapping experiment adapted from Allen et al. (2022). We used two different types of stimulus aperture, moving bars (three scans) and moving wedges and rings (three scans). We placed colorful texture patterns inside the apertures composed of different types of visual objects. These images were randomly drawn from the image dataset previously used for retinotopic mapping (Allen et al., 2022, Benson et al., 2018, Kriegeskorte et al., 2008). Mapping stimulus flickered at 3Hz on a given location in visual field. The apertures covered up to 12.4° in eccentricity. Participants were asked to fixate throughout the experiment as the retinotopic mapping stimulus moved across the visual field. They performed a color change detection task at fixation, where they were asked to make a button press when the fixation cross changed color. Each scan lasted 5 mins (300 TRs). Runs with moving bars and moving wedges and rings were interleaved.

##### Functional localizers for mapping ventral temporal cortex

Participants completed four scans of a functional localizer experiment targeting different category-selective visual cortical areas. We used an experimental protocol based on previous work that successfully delineated category-selective areas of ventral temporal cortex (https://github.com/VPNL/fLoc) (Stigliani et al., 2015). Images from a total of five different categories, bodies, characters, faces, objects, places, were shown to the participants in an event-related fMRI protocol. Body images consisted of bodies with removed heads and limbs, character images consisted of pseudowords, faces were those of adults, object images consisted of different cars, and place images were different houses. These images were overlaid on a phase-scrambled gray background that extended 10.5° in eccentricity. Each scan contained 30 distinct image presentations with each image presented for 500 ms, interspersed with blank periods. Images from different categories were shown in a randomized order in each scan. Participants were asked to fixate throughout the experiment. Each scan lasted 3 min 48 seconds (240 TRs).

##### MRI data acquisition

We collected functional and anatomical MRI data using a 3T Siemens Prisma scanner with a 64-channel head/neck coil at NYU’s Center for Brain Imaging. All functional time series data (from the main experiment, retinotopic mapping experiment, functional localizer experiment) were registered to the same anatomical scan collected during the retinotopic mapping experiment. T1-weighted anatomical images were acquired with the following scan parameters: MPRAGE: TR = 2400 ms, TE = 2.2 ms, 0.8 mm isotropic voxels, flip angle = 8°. For the main experiment, we collected 11, 13 or 15 runs (Table 1) of functional echo-planar images (EPIs) from each participant across two separate sessions. Prior to the acquisition of functional echo-planar images (EPIS), we first acquired distortion maps with opposite phase-encoding directions (AP and PA) to correct for susceptibility-induced distortions in the functional images. Functional runs in each session were acquired using a CMRR multiband EPI sequence (TR = 1000 ms, TE = 37.6 ms, 2 mm isotropic voxels, flip angle = 68°, multiband acceleration factor = 6 (Feinberg et al., 2010, Moeller et al., 2010, Xu et al., 2013)). Functional EPIs were acquired with the same scan parameters across all three experiments. Three experiments differed in the number of TRs (slice dimensions 104*x*104*x*66; the main experiment: 396 TRs, retinotopic mapping experiment: 300 TRs, functional localizer experiment: 240 TRs).

#### 2.4 Data preparation

##### Preprocessing

DICOM files were anonymized and defaced with pydeface before being transferred from the scanner to the data server. The original DICOM files were next converted to NIfTIs and organized according to Brain Imaging Data Structure (BIDS) (Gorgolewski et al., 2016) conventions using heudiconv. For preprocessing, we used fMRIPrep 20.2.7 (Esteban et al., 2019). First, T1-weighted anatomical images were bias field corrected and skull stripped. followed by tissue segmentation (CSF, white matter, gray matter) using FAST with both T1w and T2w inputs. Cortical surface reconstruction was performed with FreeSurfer’s *recon-all* (Dale et al., 1999). For functional data, we generated a reference volume, applied skull stripping, and corrected for susceptibility distortions using the AP and PA field maps. The corrected functional reference was coregistered to the anatomical image using *bbregister* (Greve and Fischl, 2009). We estimated head motion parameters relative to this reference and applied slice-timing correction with AFNI’s *3dTshift* (Cox and Hyde, 1997). The slice-time corrected data were then resampled to anatomical space in a single interpolation step that combined all spatial transformations (motion correction, distortion correction, and coregistration). Finally, the preprocessed BOLD data were projected onto each participant’s native cortical surface *(fsnative)*, and all subsequent analyses were conducted in this surface-based space.

##### Population receptive field (pRF) model

We ran a circular 2D Gaussian population receptive field (pRF) model on each vertex timeseries data from the retinotopic mapping experiment in each participant’s native brain surface. We used the *vistasoft* software (github.com/vistalab/vistasoft, Vista Lab, Stanford University), with a custom wrapper function ((github.com/WinawerLab/prfVista, New York University) to deploy the pRF models for each participant. Each vertex pRF was parameterized by a center (*x*, *y* in deg) and a size (*σ*, one standard deviation of the Gaussian, in deg) (Dumoulin and Wandell, 2008). The responses of each vertex to the moving retinotopic mapping stimuli were predicted by multiplying the Gaussian pRFs with the binarized stimulus aperture in time, then convolving it with a hemodynamic response function to account for the delay in BOLD response. For each vertex, pRF models were fit to the averaged timeseries data from three scans of two separate retinotopic mapping stimuli (moving bars and moving wedges-rings). For each vertex, the model estimated three parameters (*x*, *y*, *σ*) by minimizing the sum of squared errors between the measured responses from the averaged timeseries data and the predicted responses from the model. The fitting procedure used a coarse-to-fine approach with HRF optimization: an initial grid search over candidate pRF parameters, followed by iterative minimization with temporal decimation, then without decimation, and finally a search to optimize the hemodynamic response function parameters jointly with the pRF parameters. Goodness-of-fit was assessed using the coefficient of determination, $R^{2}=1-\frac{RSS}{TSS}$, reflecting the proportion of variance in the measured timeseries explained by the pRF model.

##### Defining V1, V2, V3, and V4

We used Neuropythy v0.12.11 (Benson and Winawer, 2018) to delineate the early visual cortical ROI boundaries on the native brain surface of each participant. First, we visualized the eccentricity and polar angle solutions of each vertex pRF on the cortical surface, centered at the occipital pole. Then following the criteria laid out in previous papers for V1-V3 (Benson et al., 2022) and V4 (Kurzawski et al., 2025, Winawer and Witthoft, 2015) based on reversals of polar angle and eccentricity solutions, we manually drew the boundaries of these areas.

##### General linear model for functional localizers

To define ventral temporal cortex in each participant, we estimated category-selective responses in visual cortex using the functional localizer experiment defined above. We ran GLMsingle (Prince et al., 2022) on the preprocessed time series data from the four localizer runs. A design matrix was constructed with five predictors corresponding to each stimulus category (faces, bodies, cars, houses, words). Beta weights were estimated for each category at each vertex and converted to percent signal change.

##### Defining ventral temporal cortex

We used FreeSurfer v7.3.2 (Fischl, 2012) to manually define a ventral temporal cortical (VTC) area in each participant’s native inflated brain surface. To define VTC, we used a combination of anatomical and functional criteria laid out in previous work (Kay and Yeatman, 2017, Rosenke et al., 2020). First, for each participant, we computed category-selective contrast maps by subtracting the mean response (percent signal change) to four categories from the response to the remaining category (e.g., faces vs. bodies, cars, houses, and words). This yielded five contrast maps per participant, one for each category. To guide ROI definition, we created an aggregated contrast map combining three category contrasts: faces vs. others, bodies vs. others, and houses vs. others. For each contrast, vertices exceeding a threshold of 1% were labeled, and the three thresholded maps were summed to create a single overlay highlighting vertices with category-selective responses. These aggregated contrast maps were used in combination with anatomical landmarks that coincide with face-, body-, and place-selective ventral cortical areas: the mid-fusiform sulcus (MFS), which separates face-selective cortex on its lateral bank from word-selective cortex on its medial bank (Grill-Spector and Weiner, 2014, Weiner et al., 2014); the occipito-temporal sulcus (OTS), which marks the lateral extent of body-selective cortex (Peelen and Downing, 2005, Schwarzlose et al., 2005); and the collateral sulcus (CoS), which marks the medial extent of place-selective cortex (Epstein and Kanwisher, 1998, Weiner et al., 2018). VTC was defined in each hemisphere as the cortical region bounded laterally by the OTS, medially by the CoS, posteriorly by the posterior transverse collateral sulcus, and anteriorly by the anterior tip of the mid-fusiform sulcus.

##### General linear model

To model the responses of each vertex on every trial in our main experiment, we ran a general linear model on each vertex time series data using GLMsingle (Prince et al., 2022). GLMsingle integrates three techniques to improve the accuracy of single-trial beta estimates: (1) for each voxel, a custom hemodynamic response function is selected from a library of candidate functions, (2) cross-validation is used to derive noise regressors from voxels unrelated to the experimental paradigm, and (3) ridge regression is applied on a voxel-wise basis to regularize beta estimates for closely spaced trials.

The single-trial GLM was run on the preprocessed time series data of each participant concatenated across two fMRI sessions. There were 319 unique stimulus conditions: 11 image categories (10 objects + 1 blank) × 29 conditions (28 noise conditions + 1 no-noise condition). The number of trials varied across participants (see Table 1). A design matrix was constructed for each run with columns corresponding to each stimulus presented during that run. For each predictor column, a 1 was entered for each TR when the stimulus was on screen.


(1) $${\hat{y}}_{i}=\left(b_{S_{1}}x_{S_{1}}+\ldots +b_{S_{319}}x_{S_{319}}\right)*HRF_{i}+N$$

where ${\overset{ˆ}{y}}_{i}$ represents the predicted time series of vertex *i*, the *b* terms are the coefficients (beta weights), and the *x* terms are indicator variables (either 0 or 1). The subscripts denote the stimulus condition, with *S*_1_ to *S*_319_ corresponding to the 319 unique stimulus conditions. All stimulus predictors were convolved with a voxel-specific hemodynamic response function (*HRF_i_*) selected from a library of candidate functions. *N* represents nuisance variables (polynomial regressors for detrending low-frequency fMRI drift and task-irrelevant noise regressors identified by GLMsingle). Estimated beta weights were converted to percent BOLD signal change by dividing by the mean signal intensity of each vertex. The resulting beta estimates were spatially smoothed on the cortical surface using FreeSurfer’s mri_surf2surf with a 3 mm full-width at half-maximum (FWHM) Gaussian kernel (Dale et al., 1999).

#### 2.5 Analysis

##### Notation

To formalize our analysis, we introduce the following notation. Let $s\in \{1,\ldots ,N_{\text{sub j}}\}$ denote a subject and r∈{V1,V2,V3,V4,VTC} denote a region of interest (ROI). The stimuli are defined by object category *c* ∈ {1, … , *C*}, noise spatial frequency *f* ∈ *F*, and noise contrast (standard deviation) σ∈Σ.

For a given subject and ROI, let $y_{t}\in R^{V}$ represent the vector of GLM beta weights for trial *t*, where *V* is the number of voxels in the ROI. We denote the set of trials corresponding to a specific condition as *T*(*c*, *σ*, *f*) . For noise-only trials, the category is undefined (or null), denoted as $T_{\text{noise}}(\sigma ,f)$. For object trials, we denote the true category of trial *t* as *c_t_*.

##### Postprocessing

Our goal is to transform the trial-wise activation maps **y***_t_* into two summary matrices per subject and ROI: a *noise-response matrix* (quantifying the response band) and an *object-decoding accuracy matrix* (quantifying the recognition band).

1. *Voxel selection*. Before generating matrices, we filtered voxels to ensure valid retinotopic coverage and responsiveness. For all ROIs, we included only voxels *v* with population receptive field eccentricities ecc*_v_* ∈ [0°, 6°]. For VTC, we applied an additional inclusion criterion, selecting only voxels where the GLM variance explained *R*^2^*_v_* > 5%.
2. *Noise-response matrix*. To measure the response band, we computed the average magnitude of the BOLD response to noise alone. For each noise condition defined by frequency *f* and contrast *σ*, we averaged the activity across all voxels and all noise-only trials:
(2) $$R(\sigma ,f)=\frac{1}{\left|T_{\text{noise}}(\sigma ,f)\right|}\underset{t\in T_{\text{noise}}(\sigma ,f)}{\sum }\left(\frac{1}{V}\overset{V}{\underset{v=1}{\sum }}y_{t,v}\right)$$
  This yields a scalar response value for each condition, resulting in a matrix $R\in R^{\left|\Sigma |\times |F\right|}$ for each ROI.
3. *Object-decoding accuracy matrix*. To measure the recognition band, we evaluated how well object identity could be decoded from neural patterns under critical-band masking. We used a correlation-based nearest-centroid classifier.
  First, we computed a *prototype* *μ_c_* for each object category *c* by averaging the voxel patterns of all noiseless trials (*σ* = 0):
(3) $$\mu_{c}=\frac{1}{\left|T(c,0,\cdot )\right|}\underset{t\in T(c,0,\cdot )}{\sum }y_{t}$$
  For decoding a test trial *t* (with noise parameters *σ*, *f*), we calculated the Pearson correlation *ρ* between the trial pattern **y***_t_* and each category prototype *μ_k_*. The predicted category ${\hat{c}}_{t}$ was the one maximizing the correlation:
(4) $${\hat{c}}_{t}=\underset{k\in \{1,\ldots ,C\}}{\text{argmax}}\rho \left(y_{t},\mu_{k}\right)$$
  When decoding noiseless trials (where the test trial would otherwise be part of the prototype), we employed a leave-one-out procedure: the test trial was excluded from the calculation of its corresponding category prototype *μ_ct_* to prevent circularity.
  Finally, we computed the decoding accuracy *A*(*σ*, *f*) as the proportion of correctly classified trials for each noise condition:
(5) $$A(\sigma ,f)=\frac{1}{\left|T(\cdot ,\sigma ,f)\right|}\underset{t\in T(\cdot ,\sigma ,f)}{\sum }I\left({\hat{c}}_{t}=c_{t}\right)$$
  where I(⋅) is the indicator function. This produced an accuracy matrix $A\in [0,1{]}^{\left|\Sigma |\times |F\right|}$.
4. *Behavioral accuracy matrix*. Analogous to the neural decoding matrix, we computed a behavioral accuracy matrix using the psychophysical data from Subramanian et al. (2023) (see github.com/ajaysub110/criticalband-masking). The behavioral accuracy $A_{\text{behav}}(\sigma ,f)$ was calculated simply as the proportion of trials for condition (*σ*, *f*) in which the subject correctly identified the object.

##### Channel fitting

We characterized the spectral tuning of visual cortex by fitting mechanistic models to the summary matrices described above. We utilized distinct modeling approaches for the noise-response and object-decoding data to account for their different signal structures.

1. *Modeling the noise-response channel*. To quantify the response band, we fit a separable model to the noise-response matrix *R*(*σ*, *f*), characterizing the BOLD amplitude as a joint function of noise contrast *σ* and spatial frequency *f*. The model assumes that the response is the product of a Naka-Rushton contrast-response function and a Gaussian spatial-frequency tuning curve, plus a baseline:
(6) $$\hat{R}(\sigma ,f)=\beta_{\text{base}}+\beta_{\text{max}}\left(\frac{\sigma }{\sigma +\sigma_{50}}\right)\text{exp}\left(-\frac{{\left({\text{log}}_{2}f-{\text{log}}_{2}\mu \right)}^{2}}{2w^{2}}\right)$$
  where *μ* and *w* are the center frequency and width of the tuning, σ_50_ is the semi-saturation contrast, and *β*_base_ and *β*_max_ scale the response magnitude. We estimated the parameters for each subject and ROI by minimizing the sum of squared errors between the model predictions and the observed BOLD responses. The bandwidth of the noise-response channel was defined as the full width at half maximum (FWHM) of the Gaussian tuning component.
2. *Modeling the object-decoding channel*. To quantify the recognition band, we fit a probabilistic model to the trial-wise decoding accuracy. We hypothesized that the impairment of object decoding is driven by the strength of the neural response to the noise. Accordingly, the input stage of this model mirrors the structure of the noise-response model described above.
  First, the channel’s sensitivity to noise spatial frequency, *S*(*f*), is modeled using the same Gaussian tuning function:
(7) $$S(f)=\text{exp}\left(-\frac{{\left({\text{log}}_{2}f-{\text{log}}_{2}\mu \right)}^{2}}{2w^{2}}\right)$$
  The effective noise drive is the product of this sensitivity and the input noise contrast: D(σ,f)=σ⋅S(f). To capture the same gain-control mechanisms observed in the BOLD response, this drive is transformed by a divisive normalization nonlinearity (Heeger, 1993):
(8) $$E(\sigma ,f)=\frac{(1+\kappa )D}{D+\kappa }$$
  where *E* represents the normalized neural response to the noise and *κ* controls the normalization strength.
  The key distinction between the two models lies in the final stage. While the noise-response model outputs the response amplitude directly, the decoding model assumes this noise activity interferes with the decision process. We therefore map the normalized noise response *E* to the probability of correct classification using a logistic function:
(9) $$P(\text{correct}|\sigma ,f)=\frac{1}{1+\text{exp}(-m(E-b))}$$
  where *m* and *b* determine the slope and threshold of the readout constraint. We fit the model parameters by minimizing the negative log-likelihood of the observed single-trial classification outcomes (correct vs. incorrect), assuming a binomial distribution.
3. *Measuring recognition bandwidth*. Unlike the response bandwidth, the recognition bandwidth was defined based on the noise power required to impair performance, providing a metric comparable to psychophysical critical-band masking. Using the fitted decoding model, we calculated a *noise power threshold* curve Θ(*f*)—defined as the noise variance (*σ*^2^) required at frequency *f* to reduce decoding accuracy to 50% of the noise-free baseline. We defined the recognition bandwidth as the full width of this threshold curve at twice the minimum power (FW2M). This metric corresponds to the spectral bandwidth at half-power sensitivity (3 dB). The same procedure was applied to the behavioral accuracy matrices to derive the psychophysical recognition bandwidth.

## Supplementary Material

Supplement 1

## Figures and Tables

**Table 1:** Trial counts per participant. Each cell shows unique stimuli × repetitions.

SID	Runs	Total trials	Scenes in noise	Noise only	Scene only	Blank
145	13	1133	280 × 3	28 × 3	10 × 19	1 × 19
135, 136	11	1307	280 × 3	28 × 9	10 × 19	1 × 25
114, 127, 141, 146, 147, 151, 185	15	1305	280 × 3	28 × 9	10 × 19	1 × 23

## Data Availability

All data and code will be released upon publication.
